# A national survey of children’s experiences and needs when attending Canadian pediatric emergency departments

**DOI:** 10.1371/journal.pone.0305562

**Published:** 2024-06-25

**Authors:** Keon Ma, Asa Rahimi, Manasi Rajagopal, Maryna Yaskina, Ran D. Goldman, Ashley Jones, Tannis Erickson, Naveen Poonai, Candice McGahern, Laura Weingarten, Bethany Lerman, Marie-Christine Auclair, Helen Wong, Lisa Hartling, Kurt Schreiner, Shannon Scott, Samina Ali, on behalf of the Pediatric Emergency Research Canada Family Needs study team

**Affiliations:** 1 Department of Pediatrics, University of Calgary, Calgary, AB, Canada; 2 Department of Pediatrics, University of Alberta, Edmonton, AB, Canada; 3 Women and Children’s Health Research Institute (WCHRI), University of Alberta, Edmonton, AB, Canada; 4 Department of Pediatrics, University of British Columbia and BC Children’s Hospital Research Institute, Division of Emergency Medicine, The Pediatric Research in Emergency Therapeutics (PRETx) Program, Vancouver, BC, Canada; 5 Department of Pediatrics and Child Health, University of Manitoba, Winnipeg, MB, Canada; 6 Departments of Paediatrics, Epidemiology & Biostatistics, Schulich School of Medicine & Dentistry, Western University, London, ON, Canada; 7 Division of Pediatric Emergency Medicine, Children’s Hospital of Eastern Ontario, Ottawa, ON, Canada; 8 Division of Pediatric Emergency Medicine, McMaster Children’s Hospital, Hamilton, ON, Canada; 9 Division of Emergency Medicine, Hospital for Sick Children, University of Toronto, Toronto, ON, Canada; 10 Centre de recherche du CHU Sainte-Justine, Montréal, QC, Canada; 11 Faculty of Health, Dalhousie University, Halifax, NS, Canada; 12 Pediatric Emergency: Advancing Knowledge (PEAK) Research Team, University of Alberta, Edmonton, AB, Canada; 13 Faculty of Nursing, University of Alberta, Edmonton, AB, Canada; University of Perugia: Universita degli Studi di Perugia, ITALY

## Abstract

**Background:**

Optimizing a child’s emergency department (ED) experience positively impacts their memories and future healthcare interactions. Our objectives were to describe children’s perspectives of their needs and experiences during their ED visit and relate this to their understanding of their condition.

**Methods:**

514 children, aged 7–17 years, and their caregivers presenting to 10 Canadian pediatric EDs completed a descriptive cross-sectional survey from 2018–2020.

**Results:**

Median child age was 12.0 years (IQR 9.0–14.0); 56.5% (290/513) were female. 78.8% (398/505) reported adequate privacy during healthcare conversations and 78.3% (395/504) during examination. 69.5% (348/501) understood their diagnosis, 89.4% (355/397) the rationale for performed tests, and 67.2% (338/503) their treatment plan. Children felt well taken care of by nurses (90.9%, 457/503) and doctors (90.8%, 444/489). Overall, 94.8% (475/501) of children were happy with their ED visit. Predictors of a child better understanding their diagnosis included doctors talking directly to them (OR 2.21 [1.15, 4.28]), having someone answer questions and worries (OR 2.51 [1.26, 5.01]), and older age (OR 1.08 [1.01, 1.16]). Direct communication with a doctor (OR 2.08 [1.09, 3.99]) was associated with children better understanding their treatment, while greater fear/ ‘being scared’ at baseline (OR 0.59 [0.39, 0.89]) or at discharge (OR 0.46 [0.22, 0.96]) had the opposite effect.

**Interpretation:**

While almost all children felt well taken care of and were happy with their visit, close to 1/3 did not understand their diagnosis or its management. Children’s reported satisfaction in the ED should not be equated with understanding of their medical condition. Further, caution should be employed in using caregiver satisfaction as a proxy for children’s satisfaction with their ED visit, as caregiver satisfaction is highly linked to having their own needs being met.

## Introduction

Family-centred care and satisfaction are essential to good medical care [1,2]. The emergency department (ED) is an inherently stressful environment for families [3], due to the unplanned nature of most visits, wait times, unpredictable clinical courses, and the high turnover of healthcare providers (HCPs) [4]. Optimizing the family experience is crucial to positively influence a child’s memories and future feelings about healthcare [5]. Within ED settings, parents have expressed that they value emotional support, coordination, eliciting and respecting preferences, involvement of the family in decision-making, timely and attentive care, information, communication, education, pain management, a child-focused environment, and continuity and transition [6]. While caregivers are often “the voice” for their children, they are not an exact proxy. For instance, a meta-analysis has shown only moderate consistency between caregivers and children reports when asked to rate pain [7].

Healthcare experiences will not improve until both children and their families play a leading role in addressing concerns identified directly by them [2]. In a study to improve caregiver communication with ED HCPs, referred to as the “Clear and Concise Communication” or “3C” initiative, communication scores improved when caregivers were given a form to elicit their concerns, but these effects dissipated once the form was no longer circulated [8]. Additional family needs studies have largely focused on disease-specific information for caregivers [9–13]. Within the inpatient setting, an Italian study found that children’s conceptualization of feeling ill was not simply linked to a medical condition, but also the “ability to form relationships with others, play, be active, and feel alive within the hospital environment” [14]. This emphasizes the importance of being attuned to a variety of psychosocial needs that children have when they present for medical care. Interestingly, a small study has previously found that audiobooks can reduce fear and anxiety for children in ED waiting rooms [15]. Other small studies of child satisfaction in the ED have focused on more general information needs and pain management [16,17].

Our primary objective was to describe children’s perspectives on their emotional, practical and general informational needs and experiences in Canadian pediatric ED settings. Our secondary objectives were to (1) compare caregiver versus child perspectives on needs and experiences during their ED visit, and (2) relate demographic characteristics and needs within the ED to a child’s understanding of their diagnosis and treatment.

## Methods

### Study design and setting

Children (and their caregivers) were recruited from 10 of 15 pediatric EDs across Canada. Sites included: Stollery Children’s Hospital (SCH) (Edmonton, AB), Alberta Children’s Hospital (Calgary, AB), BC Children’s Hospital (Vancouver, BC), Winnipeg Children’s Hospital (Winnipeg, MB), McMaster Children’s Hospital (Hamilton, ON), Children’s Hospital at London Health Sciences Center (London, ON), Children’s Hospital of Eastern Ontario (Ottawa, ON), Hospital for Sick Children (Toronto, ON), CHU Ste Justine (Montreal, QC), and IWK Health Centre (Halifax, NS).

Descriptive cross-sectional surveys were distributed to a convenience sample, over 1-week periods per season, for a total of 4 weeks over the course of one year; responses were collected from October 1, 2018-March 30, 2020, with staggered start dates. The child-specific survey was part of a larger family needs survey across Canada. Recruitment was capped at 50 caregivers per seasonal week, for a total of 200 caregivers per site, for pragmatic and cost-related reasons. This type of recruitment method has been employed in other studies to account for seasonal variation in disease presentations as well as overcrowding [18,19]. Children over the age of 7 provided assent, and completed the child survey. Only caregiver-child dyads were considered in this sub-study, leading to a smaller overall participation rate that for the full study. Ethics approvals were obtained from the Research Ethics Boards (REB) of all sites, including the University of Alberta Health REB (lead site: Pro00075437); the University of Calgary Conjoint Health REB; the University of British Columbia Children’s and Women’s REB; the Western University Health Sciences REB; the Children’s Hospital of Eastern Ontario REB; the CHU Ste Justine REB; the SickKids REB; the IWK Health Centre REB; the McMaster University REB; and the University of Manitoba Health REB. The site leads and research coordinators had access to information that could identify individual participants at their own site, only, during data collection. All data were anonymized prior to analyses and sharing of results within the research team.

### Selection of participants

Inclusion criteria were children aged 7 to 17 years with any chief complaint, and both they and their caregiver were able to read and write in either English or French. Families were excluded if, as per an HCP, (a) the child remained medically unstable throughout their ED stay, (b) there was a suspicion of child abuse, (c) the child was presenting with an altered level of consciousness, or (d) the accompanying caregiver was not a legal guardian of the child. Trained research assistants obtained written informed consent from caregivers and assent from children 7 years and older. Each participant could take part in the study only once.

### Survey development

Both the child and caregiver surveys were created and reported using published methodological guidelines and CHERRIES [20,21]. Item generation occurred through literature review, team input, and parent advisory group review. A six-member expert panel (including emergency medicine, nursing, child life services, parents, youth, and ED administrators) used the Delphi process [21] to review, rank, and reduce the items (item generation and reduction). The surveys then underwent pre-testing (8 participants contacted via email), pilot testing (10 participants in ED setting), and sensibility testing (10 participants in ED setting). The main themes of both the caregiver and the child survey included emotional, practical, and communication needs and overall satisfaction with care. The caregiver surveys had 61 questions in total, while the child survey was abbreviated and had 24 questions. Notably, our patient partners advocated for a separate child survey that matched caregiver questions, but in simpler language. (See S1 Appendix).

### Data collection

Research staff were trained by the principal investigator and national coordinator. The research assistants conducted eligibility screening. Once consent and assent were obtained, tablets were handed to children to performed data entry directly into a Research Electronic Data Capture (REDCap) database [22], a secure, online data entry system hosted at the University of Alberta. Research assistants assisted children with the technical aspects of survey completion but were instructed not to assist children in selecting responses. Children completed surveys in their ED patient room, prior to being discharged. All survey materials were provided electronically; no paper copies were employed. Caregivers were instructed not to provide suggested responses to their child. Child surveys with age-appropriate language were administered at the end of the ED visit, as national feedback from Pediatric Emergency Research Canada affirmed that this was most congruent with school-aged children’s cognitive and emotional development (concern with immediate needs, rather than delayed reflection) and significantly minimizes the loss to follow up experienced with a delayed phone call or email. Descriptive characteristics were collected from caregivers during the ED visit (the caregiver in-ED survey). Caregivers completed a follow up survey either in the ED or via an email or phone follow-up, up to 7 days post visit (the caregiver experiences survey). A 5$ gift card was provided to each family upon completion of the surveys. Participants could skip any questions they wished; missed responses were not permuted.

### Measurements

Descriptive variables from medical record review included age, sex, Canadian Triage and Acuity Scale (CTAS) score, mode of arrival, length of stay (LOS), time to see physician, interventions, and final diagnosis. Other demographic variables were obtained by family disclosure. Survey questions were administered with a Likert scale or binary responses. Several caregiver questionnaires were completed as part of the larger family needs survey.

### Analysis

Descriptive statistics for continuous variables (e.g., LOS, age) were presented as means and standard deviations or medians and quartiles. Categorical variables (e.g., sex, Canadian Triage and Acuity Scale) were presented as frequency distributions, including 5-point Likert scale questions. Likert 1–3 were considered “needs were not met”, and 4–5 was considered “needs were met”. Cohen’s kappa was used to evaluate the agreement between children and their caregivers’ assessment of privacy during exams. Multivariable logistic regression was used to ascertain effects of *a priori* selected variables (e.g., previous ED visits, language, self-reported chronic illness) on child-reported understanding of diagnoses, treatment, and caregiver perception of overall needs, and a p-value less than 0.05 was considered statistically significant. Statistical analysis was performed using SAS Ver. 9.4 (SAS Institute Inc., Cary, NC, USA). Thematic coding for free text responses (practical needs, overall care needs) was conducted by two team members (KM, AR). A coding reliability approach was employed, to identify shared topics expressed by children. Topics were not specified *a priori* but rather identified through review of responses. All responses were categorized into common topics identified independently by two team members, after which a consensus meeting was held to finalize the categorization.

## Results and discussion

### Demographic characteristics

A total of 514 child-caregiver pairs completed the survey, with a mean child age of 11.8 years (SD 3.0); 56.5% (n = 290) were female. 87.1% (n = 444) were discharged home, while 12.9% (n = 66) were admitted/transferred. Median ED length of stay was 4.1 hours (IQR 2.8–6.5). Labs or bloodwork was performed in 43.0% (221/514) of children, and 42.8% (220/514) received oral medications. Mean caregiver age was 42.9 years (SD 7.5) and 78.2% (395/505) were mothers (Tables 1 and S1).

**Table 1:** Demographic characteristics of families.

Characteristic	n (%)
**Child’s Age, years, mean (SD) (n = 513)**	11.8 (3.0)
**Child Sex, female (n = 513)** **male**	290 (56.5) 223 (43.5)
**Caregiver’s Age, years, mean (SD) (n = 491)**	42.9 (7.5)
**CTAS**^**a**^ **Score (n = 510)**	
1 –Resuscitation	2 (0.4)
2 –Emergent	85 (16.7)
3 –Urgent	269 (52.7)
4 –Semi urgent	133 (26.1)
5 –Non urgent	21 (4.1)
**Mode of arrival (n = 504)**	
Private vehicle	461 (91.5)
Ambulance (EMS)	28 (5.6)
Walked in	14 (2.8)
Public transport	1 (0.2)
**Previous ED**^**b**^ **visits (n = 504)**	
None	44 (8.7)
1–5	301 (59.7)
6–10	93 (18.5)
>10	66 (13.1)
**Previous hospitalizations (n = 506)**	
None	312 (61.7)
1–5	168 (33.2)
6–10	10 (2.0)
>10	16 (3.2)
**Length of Stay (in hours) (n = 504)**	
**Median (IQR)**	4.1 (2.8–6.5)
**Time to see physician (in hours) (n = 492)**	
**Median (IQR)**	1.4 (0.7–2.5).
**Total Number of Siblings (n = 481)**	
0	50 (10.4)
1	207 (43.0)
2	125 (26.0)
>2	99 (20.6)
**Province of Residence (n = 429)**	
Ontario	164 (38.2)
Alberta	131 (30.5)
British Columbia	53 (12.4)
Quebec	39 (9.1)
Manitoba	26 (6.1)
Nova Scotia	16 (3.7)
**Main spoken language at home (n = 504)**	
English	397 (78.8)
French	42 (8.3)
Other	65 (12.9)
**Caregiver Relationship to child (n = 505)**	
Mother	395 (78.2)
Father	95 (18.8)
Other ^c^	15 (3.0)

^a^ CTAS: Canadian Triage and Acuity Scale.

^b^ ED: Emergency department.

^c^ n = 4 sibling, n = 4 aunt/uncle, n = 2 legal guardian, n = 1 step-parent.

### Emotional needs

13.1% (67/510) of children were scared (Likert 4 or 5) when they first walked into the hospital, 10.3% (52/507) when being placed in their treatment room, and 2.6% (13/509) at discharge. A small proportion of children (6.7%, 34/509) described ‘something scary’ happening to another child in the ED (i.e., bleeding, screaming, visible injuries); 59.4% of these children told someone how they felt, which was mainly their caregiver (75.0%), and 72.2% felt better after informing someone.

78.8% (398/505) of children felt there was enough privacy during conversations with their doctor or nurse, and 78.3% (395/504) during an examination. 90.0% (362/402) of caregivers felt that their child’s privacy was respected. There was poor agreement between children and their caregivers in assessing privacy during exams (Kappa 0.11 +/- SE 0.03), with agreement lower for children aged 12 to 17 years (Kappa 0.06 +/- SE 0.05), compared to children aged 7 to 11 years (Kappa 0.16 +/- SE 0.05) (Table 2).

**Table 2:** Emotional needs.

Question	Likert Scale n(%)				
	1 (Not at all)	2	3	4	5 (Very much)
**Child Survey**					
Did you feel scared when you first walked into the hospital? (n = 510)	251 (49.2)	111 (21.8)	81 (15.9)	38 (7.5)	29 (5.7)
Did you feel scared in the waiting room? (n = 509)	326 (64.0)	105 (20.6)	44 (8.6)	30 (5.9)	4 (0.8)
Did you feel scared when you got into your room in the emergency department? (n = 507)	275 (54.2)	109 (21.5)	71 (14.0)	24 (4.7)	28 (5.5)
Do you feel scared to go home? (n = 509)	433 (85.1)	43 (8.4)	20 (3.9)	3 (0.6)	10 (2.0)
Did you feel that you had enough privacy when the doctors or nurses were talking to you? (n = 505)	44 (8.7)	21 (4.2)	42 (8.3)	89 (17.6)	309 (61.2)
Did you feel that you had enough privacy when the doctors or nurses were giving you a check-up? (n = 504)	46 (9.1)	22 (4.4)	41 (8.1)	96 (19.0)	299 (59.3)
**Caregiver Survey**					
Did you feel scared during the ED^a^ visit? (n = 400)	236 (59.0)	72 (18.0)	56 (14.0)	30 (7.5)	6 (1.5)
If so, how much did the ED^a^ team make you feel better about this? (n = 173) ^b^	28 (16.2)	20 (11.6)	42 (24.3)	45 (26.0)	38 (22.0)
Did you feel that your child’s privacy was respected? (n = 402)	6 (1.5)	6 (1.5)	28 (7.0)	83 (20.6)	279 (69.4)
Were your emotional needs (e.g. reassurance, comforted if you were upset) met by the emergency staff during your ED^a^ visit? (n = 402)	26 (6.5)	27 (6.7)	59 (14.7)	139 (34.6)	151 (37.6)

^a^ ED: Emergency department.

^b^ n = 204 did not feel scared during the emergency department visit.

### Communication needs

91.1% (451/495) and 91.4% (466/510) of children reported doctors and nurses talked directly to them, respectively. 69.5% (348/501) of children, compared to 80.2% (317/395) of caregivers, understood the diagnosis. 89.4% (355/397) of children who had tests done reported that they understood the rationale, compared to 78.5% (233/297) of their caregivers. 67.2% (338/503) of children felt they understood their treatment, compared to 80.7% (201/249) of caregivers.

84.2% (235/279) of children felt someone answered their questions and worries. Children reported nurses (36.3%, 139/383), doctors (34.2%, 131/383), parents (27.7%, 106/383) and other healthcare professionals (1.5%, 6/383) responded to their questions (Table 3).

**Table 3:** Communication needs.

**Child Survey**					
**Question**	**No n(%)**		**Yes n(%)**		
Did the nurse(s) talk directly to you (and not just your parents)? (n = 510)	44 (8.6)		466 (91.4)		
Did the doctor(s) talk directly to you (and not just your parents)? (n = 495)	44 (8.9)		451 (91.1)		
Do you understand what is wrong with you (your diagnosis)? (n = 501)	153 (30.5)		348 (69.5)		
Do you understand why the doctor did tests on you? (n = 397) ^a^	42 (10.6)		355 (89.4)		
Do you understand what will make you better? (n = 503)	165 (32.8)		338 (67.2)		
Did someone answer your questions and/or worries? (n = 279) ^b^	44 (15.8)		235 (84.2)		
**Caregiver Survey**					
	**Likert Scale n(%)**				
**Question**	**1 (Very Bad /Little)**	**2**	**3**	**4**	**5 (Very Good /Much)**
How was the overall communication between you and your child’s nurse(s)? (n = 398)	12 (3.0)	17 (4.3)	42 (10.6)	114 (28.6)	213 (53.5)
How was the overall communication between you and your child’s doctor(s)? (n = 398)	7 (1.8)	13 (3.3)	37 (9.3)	131 (32.9)	210 (52.8)
Did the doctors, nurses and other providers involve your child in their own care? (n = 397)	8 (2.0)	14 (3.5)	40 (10.1)	117 (29.5)	218 (54.9)
How clear was the information provided to you about your child’s condition? (n = 395)	14 (3.5)	17 (4.3)	49 (12.3)	116 (29.2)	201 (50.6)
How clear was the information provided to you about your child’s tests done in the emergency department? (n = 297) ^c^	6 (2.0)	16 (5.4)	42 (14.1)	66 (22.2)	167 (56.2)
How clear was the information provided to you about any medicines your child received? (n = 249) ^d^	11 (4.4)	9 (3.6)	28 (11.2)	64 (25.7)	137 (55.0)
Did the emergency staff answer your questions and concerns? (n = 399)	9 (2.3)	20 (5.0)	51 (12.8)	127 (31.8)	192 (48.1)
How satisfied were you with the information given to you before being discharged/admitted? (n = 397)	19 (4.8)	18 (4.5)	57 (14.4)	134 (33.8)	169 (42.6)

^a^ n = 105 did not receive any tests in the emergency department.

^b^ n = 229 did not have any questions or worries.

^c^ n = 98 answered N/A for information provided around their child’s tests.

^d^ n = 146 answered N/A for the clarity of information provided about medicines their child received.

### Practical needs

82.4% (420/510) of children felt the ED was clean, while 42.5% (217/510) felt it was quiet. Of those allowed to eat and drink, 36.5% (143/392) reported being shown where to get food and drinks.

The top three reported items to make children happier while waiting were electronics (i.e., video games, tablet, phone) (50.0%, 175/350), non-electronic entertainment (i.e., toys, crafts, books) (28.9%, 101/350), and food and drink (15.1%, 53/350). Children prioritized items such as pillows and blankets (23.7%, 76/321), and entertainment (i.e., tablets, WiFi, books, toys) (19.9%, 64/321) as what would make them more comfortable while waiting; 40.8% (131/321) of children said “nothing else” could make them more comfortable.

### Overall care and experience

43.1% of children (217/504) and 38.2% (152/398) of caregivers felt they waited too long to see a doctor. 90.9% (457/503) of children felt well taken care of by nurses, and 90.8% (444/489) by doctors; 94.8% (475/501) of children were happy at the end of their ED visit. From their caregivers’ perspectives, 79.7% (310/389) felt their child’s needs were met; 77.5% (299/386) felt their own needs were met during the visit (Table 4).

**Table 4:** Perspectives on overall care.

**Child Survey**					
**Question**	**Likert Scale n(%)**				
	**1** **(Not well)**	**2**	**3**	**4**	**5** **(Very well)**
How well did the nurse(s) take care of you today? (n = 503)	5 (1.0)	10 (2.0)	31 (6.2)	109 (21.7)	348 (69.2)
How well did the doctor(s) take care of you today? (n = 489)	5 (1.0)	8 (1.6)	32 (6.5)	106 (21.7)	338 (69.1)
**Caregiver Survey**					
**Question**	**1** **(Not at all)**	**2**	**3**	**4**	**5** **(Very much / Very well)**
Overall, to what extent did we meet your child’s needs? (n = 389)	13 (3.3)	17 (4.4)	49 (12.6)	113 (29.0)	197 (50.6)
Overall, to what extent did we meet your needs? (n = 386)	13 (3.)	22 (5.7)	52 (13.5)	127 (32.9)	172 (44.6)
Was your child’s pain well managed? (n = 385)	21 (5.5%)	29 (7.5)	51 (13.2)	96 (24.9)	188 (48.8)

When children were asked to describe what went well during the ED visit, the top two responses were good assessment/management of their condition (35.9%, 129/359) and friendly staff who provided emotional support (35.7%, 128/359). 16.4% (59/359) of children said “everything” went well. When asked what could be done differently, the top two responses were shorter wait times (23.1%, 80/346) and practical needs including food and toys (14.7%, 51/346); 26.0% (90/346) said “nothing else” was needed (S2 Table).

### Logistic regression analyses

Predictors of a child’s self-reported better understanding of their diagnosis included doctors speaking directly to the child (OR 2.21, 95% Cl 1.15, 4.28), having someone answer a child’s questions or worries (OR 2.51, 95% Cl 1.26, 5.01), and older child age (OR 1.08 per year, 95% Cl: 1.01, 1.16). Direct communication with nurses, feeling scared on presentation or discharge, prior hospitalizations, and main language at home were not shown to significantly influence a child’s understanding of their diagnosis (S3 Table).

Predictors of a child’s self-reported better understanding of their own treatment included doctors speaking directly with the child (OR 2.19, OR 1.13, 4.24), while feeling more scared when walking into the hospital (OR 0.77, 95% CI 0.66, 0.90) or at discharge (OR 0.46, 95% Cl 0.22, 0.96) had the reverse effect. A child’s age, prior hospitalizations, main language at home, having a chronic illness, and direct communication with nurses or having someone answer their questions or worries were not associated with better understanding of their treatment (S4 Table).

The greatest predictors of a caregiver’s perceptions of their child’s needs being met included their own needs being met (OR 23.76, 95% CI 15.02, 37.59), adequate pain management (OR 6.51, 95% Cl 4.20, 10.11), a caregiver emotional needs being met by staff (OR 1.76, 95% Cl 1.11, 2.78), and a child being involved in their own care (OR 1.72, 95% Cl 1.08, 2.74). Caregivers of older children had a lower likelihood of perceiving their child’s needs being met (OR 0.94 per year, 95% Cl 0.90, 0.99) (S5 Table).

## Discussion

In this Canada-wide, patient-partnered study of children’s perspectives on their needs and experiences during pediatric ED visits, almost all children were happy and felt well taken care of. However, close to one-third of children did not understand their diagnosis or treatment. Children who were less scared and had someone address their worries better understood their diagnosis and treatment. Further, caregivers’ perspectives were not always representative of their child’s, particularly for privacy needs during a healthcare encounter.

Having a physician speak directly to a child was predictive of greater understanding in our study and underscores the importance of direct communication with a child during healthcare encounters. This suggests that children’s emotional needs may be closely intertwined with their capacity to understand, as feeling less scared walking into the hospital (baseline) and at discharge were both predictive of greater understanding of their treatment. In our study, a higher proportion of children expressed “feeling scared” when arriving at the hospital and when being placed in the treatment room compared to other time points during the visit. While there may be many factors contributing to these feelings, HCPs should recognize these periods as an opportunity to provide additional emotional support, as required. It is known that stress/fear negatively impacts the ability to learn and retain information in both children and youth [23,24]. Effective communication with children involves more than speaking directly to them, and our findings suggest that improving a child’s emotional safety enables better understanding and retention of information, and is of particular importance in the ED, which is inherently stressful and sometimes traumatic [3]. A prior study interviewing preschoolers found that their conceptualization of illness had a high degree of concrete-operational explanations, which is more typical of children aged seven to ten years. This suggests that we may often underestimate children’s capacity to understand medical information. A larger-scale qualitative study could serve to better clarify the paradigms through which children, of varying developmental stages, understand what brings them to the ED and the treatments they receive [25].

Despite some children reporting a poor understanding of their condition in our study, almost 95% reported high satisfaction with their ED visit. In contrast, approximately 80% of caregivers reported their child’s needs were addressed during the visit, and just over three-quarters felt their own needs were met during the visit. Caregivers were almost 24 times as likely to perceive their child’s needs as met if their own needs were met during the visit; this could indicate that caregivers may have trouble disentangling their child’s needs being met from their own needs being met. Although this paper is not specifically focused on caregiver satisfaction, it is known that caregiver expectations around diagnostic tests, antibiotic use, or subspecialty consultation (even when not indicated) can influence their satisfaction [26]. There is a need to explore the role of cultural influences and the impact of expectation setting by physicians outside EDs in caregiver satisfaction. Caregiver trust was also not specifically assessed in this study. Most caregivers do not have pre-existing therapeutic alliances with ED HCPs. While HCPs may attempt their best at an individual level to establish rapport with both caregiver and child, broader societal mistrust of the healthcare system may also play into their satisfaction with a visit [27]. As existing research around satisfaction focuses primarily on caregivers, our findings also highlight the need for future studies to address children directly.

In our study, under 80% of children felt their privacy was respected in conversations and exams, compared to 90% of caregivers. This is congruent with a prior single centre study in which parents also underestimated their child’s need for privacy [28]. There was almost no agreement between children and their caregivers for privacy needs during exams, and this remained true regardless of child age. Our results suggest that we may be under-estimating the need for privacy for younger children. Children of all ages should be asked who they would like in the room during exams and the clinical environment should be optimized to preserve privacy.

## Conclusion

While almost all children were happy with their ED visit, close to one-third lacked understanding of their diagnosis and treatments received. Children’s reported satisfaction should not be equated with understanding their medical care. Improving a child’s sense of emotional safety is linked to better comprehension of information presented. Clinicians and researchers should be cautious in using caregiver satisfaction as a proxy for children’s satisfaction with their ED visit, as caregiver satisfaction is highly linked to having their own needs being met. Caregivers may overestimate or misread their child’s comfort, particularly as it relates to privacy needs. Understanding the interplay and differences between child and caregiver needs and satisfaction is critical to optimizing child-patient experience within EDs and the broader healthcare system.

### Limitations

Due to logistical constraints, we were only able to provide this survey in English and French. Our patient partners have pointed out the need for a future inclusive study that captures families from more diverse linguistic and cultural backgrounds, and a larger representation of those from lower household income or educational attainment. The survey was administered during day and evening hours with research assistant coverage, so it may not have captured needs across the overnight period in the ED. The caregiver experiences portion of the survey could be completed up to 7 days after their ED visit, potentially introducing recall bias to our study and be confounded by additional experiences following their ED visit. Further, as the child survey results presented in this manuscript were a sub-study of a larger caregiver-focused study, the generalizability of our results may be affected. This was, however, partially mitigated by sampling 10 of the 15 pediatric EDs in Canada. This survey may in and of itself exclude families who are too emotionally burdened or stressed to participate in the moment and miss capturing the unique needs of families in the highest stress situations.

## Supporting information

S1 Appendix Survey tool. (PDF)

S1 Table Additional demographic characteristics of families. (DOCX)

S2 Table Thematic coding. (DOCX)

S3 Table Univariable model for a child’s understanding of their diagnosis. (DOCX)

S4 Table Univariable model for a child’s understanding of their treatment. (DOCX)

S5 Table Univariable model for a caregiver’s perception of meeting their child’s needs. (DOCX)
