# Supplementary material for: A national survey of children’s experiences and needs when attending Canadian pediatric emergency departments
Source: PLoS One. 2024 Jun 25;19(6):e0305562. doi: 10.1371/journal.pone.0305562 (PMC11198794; doi:10.1371/journal.pone.0305562)
Supplement: S1 Appendix — (PDF) [file pone.0305562.s001.pdf]

Screening & Contact Information

Record ID ______________________

Note: ONLY enrolled participants should be entered into the REDCap database.

Inclusion Criteria (should be YES to all)

	Yes	No
1. Caregiver of child aged 0-17 years	◯	◯
2. Child presenting to 1 of 11 participating Pediatric Emergency Departments	◯	◯
3. Ability to read and write English (or French, for Quebec and Ontario)	◯	◯

Caregiver is ineligible for the study. Inclusion criteria must all be 'Yes' for the caregiver to be eligible.

Exclusion Criteria (should be NO to all)

	Yes	No
1. The child remains medically unstable throughout their PED stay, as determined by the healthcare providers	◯	◯
2. There is suspicion of child abuse, as determined by the healthcare provider	◯	◯
3. The child is presenting with an altered level of consciousness	◯	◯
4. The accompanying parent is not a legal guardian of the child	◯	◯

Caregiver is ineligible for the study. Exclusion criteria must all be 'No' for the caregiver to be eligible.

Caregiver is eligible for the study. Please proceed with consenting the caregiver.

Child Age

Is the patient 7 years of age, or older? ◯ Yes ◯ No

Child is ineligible to complete the study survey. However, you may proceed with consenting the caregiver.

Child is eligible to complete the study survey. Please proceed with assenting the child.

Informed Consent / Assent

Was consent obtained from the parent / caregiver? ◯ Yes ◯ No

Was assent obtained from the child? ◯ Yes ◯ No

Family should not be enrolled in the study. Legal guardian must consent.

Contact Information

Child Name

____
(First & Last Name)

Parent/ Caregiver Name

____
(First & Last Name)

Preferred Method of Follow-up:
◯ Email
◯ Phone

Email

____
(Parent / Caregiver Email)

Phone Number

____
(Parent / Caregiver Phone Number)

Alternate Phone Number

____

Preferred time to call

____

In Pediatric Emergency Department Caregiver Survey

Thank you for taking the time to complete this questionnaire. Information from the survey will be used for research purposes only and will be viewed only by members of the research team. No identifying information, including your name, will be used in any report or publication.

These questions ask about your and your child's demographics. In addition, we will ask you to look at some general health information and then answer a few questions about that information. If there are any survey questions that you would prefer not to answer, you are free to skip them.

Your responses will not affect your child's care in the emergency department.

Thank you for your participation!

Your Child's Information

Why did you bring your child to the emergency department today?

- ◯ Child's own doctor not available
- ◯ Sent in by a doctor
- ◯ No Family doctor / Primary care doctor
- ◯ Telephone advice line said to come in
- ◯ I thought we needed Emergency Department treatment
- ◯ Other (please specify)

Please Specify: __________________________________

How many times before has your child been seen in an emergency department?

- ◯ Never. This is the first time.
- ◯ 1-5
- ◯ 6-10
- ◯ >10

How many times has your child been hospitalized overnight?

- ◯ None
- ◯ 1-5
- ◯ 6-10
- ◯ >10

Do you feel that your child uses more health care than is usual for most children of their age? (e.g. asthma, diabetes, cystic fibrosis)

- ◯ Yes
- ◯ No
- ◯ Unsure

Is your child visiting today because of an ongoing health issue that has worsened?

- ◯ Yes
- ◯ No
- ◯ Unsure

Your Information

What is your relationship to the child being seen in the emergency department today?

- ◯ Mother
- ◯ Father
- ◯ Grandfather
- ◯ Grandmother
- ◯ Other (please specify)

Please Specify: __________________________________

What is YOUR age, in years?

____

How many other children do you have?

- 0
- 1
- 2
- 3
- 4
- 5
- 6
- 7
- 8
- 9
- 10

What is your home postal code? (1st 3 digits only)

____

What is the main language you speak at home?

- English
- French
- Spanish
- Mandarin Chinese
- Cantonese
- Hindi/ Urdu/ Punjabi
- Arabic
- Somali
- Portuguese
- Russian
- German
- Italian
- Dutch/Afrikaans
- Other (please specify)

Please Specify:

____

What is your highest level of Education?

- Elementary School
- High School or some High School
- Diploma/Certificate
- Some Post-Secondary/University
- University/Professional Degree
- Decline to answer

What is your annual household income from all sources?

- Less than or equal to $25,000
- $25,001 to $50,000
- $50,001 to $75,000
- $75,000 to $100,000
- Greater than $100,000
- Decline to answer

Stait-Trait Anxiety Inventory

We would ask that you complete the following questions as they relate to your feelings about being in the emergency departments right now and having a child who is sick. A number of statements which people have used to describe themselves are given below. Read each statement and then circle the appropriate number to indicate how you feel right now, that is at this moment. There are no right or wrong answers. Do not spend too much time on any one statement but give the answer which seems to describe your feelings best.

		1 Not at all	2 Somewhat	3 Moderately so	4 Very much so
1.	I feel calm	◯	◯	◯	◯
2.	I feel secure	◯	◯	◯	◯
3.	I am tense	◯	◯	◯	◯
4.	I feel strained	◯	◯	◯	◯
5.	I feel at ease	◯	◯	◯	◯

		1 Not at all	2 Somewhat	3 Moderately so	4 Very much so
6.	I feel upset	◯	◯	◯	◯
7.	I am presently worrying over possible misfortunes	◯	◯	◯	◯
8.	I feel satisfied	◯	◯	◯	◯
9.	I feel frightened	◯	◯	◯	◯
10.	I feel comfortable	◯	◯	◯	◯

		1 Not at all	2 Somewhat	3 Moderately so	4 Very much so
11.	I feel self-confident	◯	◯	◯	◯
12.	I feel nervous	◯	◯	◯	◯
13.	I am jittery	◯	◯	◯	◯
14.	I feel indecisive	◯	◯	◯	◯
15.	I am relaxed	◯	◯	◯	◯

		1 Not at all	2 Somewhat	3 Moderately so	4 Very much so
16.	I feel content	◯	◯	◯	◯
17.	I am worried	◯	◯	◯	◯
18.	I feel confused	◯	◯	◯	◯
19.	I feel steady	◯	◯	◯	◯
20.	I feel pleasant	◯	◯	◯	◯

As a part of this study, we are measuring something called 'Health Literacy', or a person's general understanding of health information. For this, we ask you to please answer the following questions about a Food Label.

Nutrition Facts

Serving Size 1/2 cup (125 mL)
Servings Per Container 4

Amount Per Serving	% Daily Value*
**Calories** 250	
**Fat** 13 g	**20** %
Saturated 9.0 g + Trans 0 g	**45** %
**Cholesterol** 30 mg	
**Sodium** 55 mg	**2** %
**Carbohydrate** 30 g	**10** %
Fibre 0 g	**0** %
Sugars 23 g	
**Protein** 4 g	

Vitamin A	10 %	Vitamin C	0 %
Calcium	15 %	Iron	4 %

* Percentage Daily Values are based on a 2,000 Calorie diet. Your daily values may be higher or lower depending on your Calorie needs.

INGREDIENTS: Cream, skim milk, liquid sugar, water, egg yolks, brown sugar, milkfat, peanut oil, sugar, butter, salt, carrageenan, vanilla extract

If you eat the entire container, how many calories will you eat?

◯ 250 Calories ◯ 300 Calories ◯ 500 calories ◯ 1000 Calories ◯ I do not know

If you are allowed to eat 60 grams of carbohydrates as a snack, how much ice cream could you have?

◯ 1⁄2 cup (125 mL) ◯ 1 cup (250 mL) ◯ 2 cups (500 mL) ◯ 4 cups (1000 mL) ◯ I do not know

Your doctor advises you to reduce the amount of saturated fat in your diet. You usually have 42 g of saturated fat each day, which includes one serving of ice cream. If you stop eating ice cream, how many grams of saturated fat would you be eating each day?

◯ 9 grams ◯ 15 grams ◯ 33 grams ◯ 42 grams ◯ I do not know

If you usually eat 2,500 calories in a day, what percentage of your daily value of calories will you be eating if you eat one serving of ice cream?

◯ 5% ◯ 10% ◯ 20% ◯ 250% ◯ I do not know

Pretend that you are allergic to the following substances: penicillin, peanuts, latex gloves and bee stings. Is it safe for you to eat this ice cream?

◯ Yes ◯ No ◯ I do not know

Why not?

◯ Contains egg ◯ Comes from bees ◯ Contains peanut or peanut oil ◯ Contains ingredients that may be harmful ◯ I do not know

NVS Score

Score of 0-1 suggests high likelihood (50% or more) of limited literacy.
Score of 2-3 indicates the possibility of limited literacy.
Score of 4-6 almost always indicates adequate literacy.

____
(0-6)

In Pediatric Emergency Department Child Survey

Assent not obtained; Do not complete survey with child.

Child under the age of 7 years; Do not complete survey with child.

Emotional Needs

These questions are for your emergency visit today. Please answer the following questions, where 1 means 'not at all' and goes all the way to 5, which means 'very much'.

		1 (Not at all)	2	3	4	5 (Very much)
1.	Did you feel scared when you first walked into the hospital?	◯	◯	◯	◯	◯
2.	Did you feel scared in the waiting room?	◯	◯	◯	◯	◯
3.	Did you feel scared when you got into your room in the emergency department?	◯	◯	◯	◯	◯
4.	Right now, do you feel scared to go home?	◯	◯	◯	◯	◯
5.	Did you feel that you had enough privacy when the doctors or nurses were talking to you?	◯	◯	◯	◯	◯
6.	Did you feel that you had enough privacy when the doctors or nurses were giving you a check-up?	◯	◯	◯	◯	◯

7. Did you see something scary happen to another child in the emergency department? ◯ Yes ◯ No

If yes, what happened? ______________________________

Did you tell anyone about how you felt? ◯ Yes ◯ No

Who did you tell?
- ☐ Mom/Dad
- ☐ Nurse
- ☐ Doctor
- ☐ Other (please specify)

Please Specify: ______________________________

	1 (Not at all)	2	3	4	5 (Very much)
How much did they help you with feeling better?	◯	◯	◯	◯	◯

Information Needs

1. Did the nurse(s) talk directly to you? (and not just your parents) — ◯ Yes ◯ No
2. Did the doctor(s) talk directly to you? (and not just your parents) — ◯ Yes ◯ No
3. Do you understand what is wrong with you (your diagnosis)? — ◯ Yes ◯ No
4. Do you understand why the doctor did tests on you? — ◯ Yes ◯ No ◯ I didn't have any tests
5. Do you understand what will make you better? — ◯ Yes ◯ No

Emergency Operations

1. Did you have to wait a long time to see a doctor?
   - ◯ Yes, it was too long
   - ◯ No, it was okay
2. What would make you happier while waiting? ______________________________
3. Did someone answer your questions and/or worries?
   - ◯ Yes
   - ◯ No
   - ◯ I did not have any questions or worries

If Yes, who answered them for you?
- ☐ Mom/Dad
- ☐ Nurse
- ☐ Doctor
- ☐ Other (please specify)

Please Specify: ______________________________

Practical Needs

These questions are for your emergency visit today. Please answer the following questions, where 1 means 'not at all' and goes all the way to 5, which means 'very much'.

		1 (Not at all)	2	3	4	5 (Very much)
1.	How clean is the emergency department?	◯	◯	◯	◯	◯
2.	How quiet is the emergency department?	◯	◯	◯	◯	◯

3. If you were allowed to eat, did someone show you where to get food and drinks in the emergency department?
   - ◯ Yes
   - ◯ No
   - ◯ I was not allowed to eat and drink

4. What other things did you need, today, to make you more comfortable in the emergency department?

____

Overall Care

	1 (Not at all)	2	3	4	5 (Very much)
1. How well did the nurse(s) take care of you today?	◯	◯	◯	◯	◯
2. How well did the doctor(s) take care of you today?	◯	◯	◯	◯	◯

3. Overall, did we make you happy today? ◯ Yes ◯ No

4. What did we do well today?

____

5. What is the ONE most important thing you would tell us to do differently, if you came to the emergency department again?

____

Medical Record Review (to be completed by RA)

Child Demographics & Visit Details

Age

____
(years)

Sex
- ○ Male
- ○ Female

Triage Date & Time

____
(dd-mm-yyyy HH:MM)

CTAS
- ○ 1 - Resuscitation
- ○ 2 - Emergent
- ○ 3 - Urgent
- ○ 4 - Semi urgent
- ○ 5 - Non urgent

Mode of Arrival
- ○ EMS
- ○ Private vehicle
- ○ Other

Specify other mode of arrival:

____

Date & Time first seen by a Physician

____
(dd-mm-yyyy HH:MM)

Co-interventions Received

(check all that apply)
- ☐ IV insertion
- ☐ Labs
- ☐ X-ray
- ☐ Consultation with another specialty
- ☐ Oral medications
- ☐ IV medications
- ☐ Procedural sedation
- ☐ Cast application
- ☐ In-ED procedure (laceration repair, lumbar puncture)
- ☐ Other (specify below)

Specify other co-interventions received in the ED:

____

Date & Time of Discharge from the ED

____
(dd-mm-yyyy HH:MM)

Discharge disposition
- ○ Discharged
- ○ Admitted
- ○ Transferred
- ○ Other (specify below)

Specify other discharge disposition:	__________________________________
Final Diagnosis<br><br>(check all that apply)	☐ Cardio-vascular (eg. myocarditis, cardiac syncope, congestive heart failure, arrhythmia)<br>☐ Head and neck (eg. otitis media, mastoiditis)<br>☐ Respiratory (eg. croup, asthma, RAD, bronchiolitis, URTI, LRTI)<br>☐ Gastro-intestinal (eg. gastroenteritis, constipation, abdominal pain)<br>☐ Genito-urinary (eg. UTI, STI, urinary retention)<br>☐ Musculoskeletal (eg. fracture, sprain, strain)<br>☐ Neurologic (eg. migraine, concussion, weakness, irritability, meningitis, vasovagal syncope, GBS)<br>☐ Skin issues (eg. cellulitis, acne, rashes)<br>☐ Other (please specify below)
Specify other final diagnosis:	__________________________________

Follow-up Call Log

Number of Follow-up Calls attempted:	◯ N/A completed by email<br>◯ 1<br>◯ 2<br>◯ 3<br>◯ 4<br>◯ 5
Date and Time of Call #1	______________________________
Date and Time of Call #2	______________________________
Date and Time of Call #3	______________________________
Date and Time of Call #4	______________________________
Date and Time of Call #5	______________________________

Follow-up Caregiver Survey

Emotional Needs

The following questions pertain to your child's visit to the emergency department a few days ago.

Please describe how well the following needs were met using a 5-point scale (1= very poor/very little, up to 5= very well/very much).

		1 (very little)	2	3	4	5 (very much)
1.	Were your emotional needs (e.g. reassurance, comforted if you were upset) met by the emergency staff during your emergency department visit?	○	○	○	○	○

2. Which healthcare provider provided the best emotional support to you?

- ○ Waiting room nurse
- ○ Bedside nurse
- ○ Trainee doctor (medical student/resident)
- ○ Doctor in charge/Senior doctor
- ○ Social worker
- ○ Other (please specify)
- ○ No one

Please Specify: __________________________________

		1 (very little)	2	3	4	5 (very much)
3.	Did you feel that your child's privacy was respected?	○	○	○	○	○

4. Did you see a traumatic or upsetting situation for another child while in the emergency department today? (e.g. child getting CPR, having a seizure) ○ Yes ○ No

If yes, please describe. __________________________________

		1 (very little)	2	3	4	5 (very much)
5.	Did you wonder whether you should have come to the hospital sooner?	○	○	○	○	○

	1 (very little)	2	3	4	5 (very much)	N/A
If so, how much did the emergency department team make you feel better about this?	○	○	○	○	○	○

		1 (very little)	2	3	4	5 (very much)
6.	Did you feel scared during the emergency department visit?	○	○	○	○	○

What scared you? __________________________________

	1 (very little)	2	3	4	5 (very much)	N/A
7. If so, how much did the emergency department team make you feel better about this?	○	○	○	○	○	○

8. Did you feel, at any time, that you were treated poorly/differently, based on your race, religion, sexual orientation, ability to speak English/French, a disability, or other reason?

○ Yes ○ No

If yes, please specify:

____

Medical Information Needs

Please describe how well the following needs were met using a 5-point scale (1= very poor/very little, up to 5= very well/very much)

		1	2	3	4	5
1.	How was the overall communication between you and your child's nurse(s)?	○	○	○	○	○
2.	How was the overall communication between you and your child's doctor(s)?	○	○	○	○	○
3.	Did the doctors, nurses and other providers involve your child in their own care?	○	○	○	○	○
4.	Did the doctors, nurses and other providers involve YOU in your child's care?	○	○	○	○	○
5.	How clear was the information provided to you about your child's condition?	○	○	○	○	○

		1	2	3	4	5	My child did not receive any tests
6.	How clear was the information provided to you about your child's tests done in the emergency department?	○	○	○	○	○	○

		1	2	3	4	5	My child did not receive any medicines
7.	How clear was the information provided to you about any medicines your child received?	○	○	○	○	○	○

		1	2	3	4	5
8.	How satisfactory were the updates to you about your child's care while you were in the emergency department?	○	○	○	○	○
9.	Did the emergency staff answer your questions and concerns?	○	○	○	○	○
10.	How satisfied were you with the information given to you before being discharged/admitted?	○	○	○	○	○

	1	2	3	4	5	My child is still in hospital
11. How comfortable were you to care for your child's injury/illness at home, after being sent home from the hospital?	○	○	○	○	○	○

Emergency Operations Information

1. Do you wish you had a translator to help you communicate during your emergency visit?
   - ◯ Yes
   - ◯ No

   If yes, please comment: __________________________________

2. Did you have to wait a long time to see a doctor?
   - ◯ Yes, it was too long
   - ◯ No, it was okay

3. Did you know how long you were going to have to wait?
   - ◯ Yes
   - ◯ No

4. What would make wait times easier for your family, besides lowering them? __________________________________

	1 (not satisfied)	2	3	4	5 (very satisfied)
5. How satisfied were you with the overall length of your emergency visit?	◯	◯	◯	◯	◯

6. If you could give any advice on how to improve the emergency department, what would you tell us? __________________________________

Overall Practical Needs

Please RANK each of these items as to how important you think they are during your emergency department visit (i.e. 1=most important, 4 = least important).

	1	2	3	4
Free/low cost parking	○	○	○	○
Clean emergency department	○	○	○	○
Safe emergency department	○	○	○	○
Quiet emergency department	○	○	○	○

Practical Needs throughout the Visit

Please RANK each of these items as to how important you think they are during your emergency department visit (i.e. 1=most important, 6 = least important).

	1	2	3	4	5	6
Access to phone	○	○	○	○	○	○
Access to electrical outlet to charge personal devices (Phones, iPads, etc.)	○	○	○	○	○	○
Access to power cords to charge personal devices (Phones, iPads, etc.)	○	○	○	○	○	○
WiFi/internet Access	○	○	○	○	○	○
Access to free food	○	○	○	○	○	○
Access to free drinks	○	○	○	○	○	○

Practical Needs in the Waiting Room

Please RANK each of these items as to how important you think they are during your emergency department visit (i.e. 1=most important, 4 = least important).

	1	2	3	4
Breastfeeding space in the waiting room	○	○	○	○
Age appropriate toys/games/books for your child in the waiting room	○	○	○	○
Video Games/TV Screens in the waiting room	○	○	○	○
Space for your family in the waiting room	○	○	○	○

Practical Needs in the Patient Room

Please RANK each of these items as to how important you think they are during your emergency department visit (i.e. 1=most important, 6 = least important).

	1	2	3	4	5	6
Toiletries/Wash Kit	○	○	○	○	○	○
Availability of diapers	○	○	○	○	○	○
Dim lighting for resting/sleeping child	○	○	○	○	○	○
Age appropriate toys/games/books for your child when in the patient bed	○	○	○	○	○	○
Video Games/TV Screens in the patient room	○	○	○	○	○	○
Size of patient room	○	○	○	○	○	○

Overall Care and Future Directions

Please describe how well we met your needs today, using a 5-point scale (1= very poor/not satisfied, up to 5= very well/very satisfied)

		1	2	3	4	5
1.	Overall, to what extent did we meet your child's needs?	○	○	○	○	○
2.	Overall, to what extent did we meet your needs?	○	○	○	○	○
3.	Was your child's pain well managed?	○	○	○	○	○

4. What could have been done differently to manage it better?

____

5. Which need was best met during your child's visit?

____

6. Which need was most poorly met during your child's visit?

____

7. Overall, what are the most important things to improve in the emergency department?

(Choose all that apply)

☐ Better use of technology
☐ Better pain management
☐ Faster diagnosis
☐ Better communication of wait times and any delays
☐ Shorter wait times
☐ Other (please specify)

Please Specify:

____

8. Is there any information about the emergency department that you wished you'd have known before arriving at the emergency department?

____

9. How do you like to receive written discharge information?

○ Paper handout
○ Email
○ Text
○ Website
○ Other (please specify)

Please Specify:

____

Thoughts on Emergency Research

1.	Are you interested in helping us to improve the health care system by participating in further research?	◯ Yes<br>◯ No
	If so, which of these options would you prefer?	◯ I want to hear about all the studies and decide for myself which studies I want my child to participate in<br>◯ I would like to be approached on an individual basis only if the study applies to my child's condition
2.	When research is done in the emergency department, what is the best time to ask your family to participate?	◯ Waiting room<br>◯ After being seen by a doctor<br>◯ Right before you can leave<br>◯ Other (please specify)
	Please Specify:	__________________________
3.	What is the best way to encourage families to participate in research?	__________________________________

Early Withdrawal Form

Date & Time of Withdrawal ______________________________

Reason for Withdrawal (select all that apply)
- ☐ Withdrawal of consent / assent
- ☐ Other, specify

Other, specify ______________________________

If withdrew consent/ assent:

	Yes	No
1. Permission to use collected data?	○	○
2. Permission to conduct Chart Review?	○	○
3. Follow-up to continue?	○	○
