# Supplementary material for: A national survey of children’s experiences and needs when attending Canadian pediatric emergency departments
Source: PLoS One. 2024 Jun 25;19(6):e0305562. doi: 10.1371/journal.pone.0305562 (PMC11198794; doi:10.1371/journal.pone.0305562)
Supplement: S1 Table — (DOCX) [file pone.0305562.s002.docx]

S1 Table. Additional Demographic Characteristics of Families

| Characteristic | n (%) |
| --- | --- |
| **Reason for visit (n=500)** | |
| I thought we needed Emergency Department treatment | 294 (58.8) |
| Sent in by a doctor | 121 (24.8) |
| Child’s own doctor not available | 37 (7.4) |
| Telephone advice line said to come in | 20 (4.0) |
| Out of home emergency, third party decision | 9 (1.8) |
| No family doctor / primary care doctor | 6 (1.2) |
| Follow up, PRN^a^, caregiver discretion | 5 (2.0) |
| **Chronic disease (n=504)** | |
| No | 376 (74.6) |
| Yes | 92 (18.3) |
| Unsure | 36 (7.1) |
| Visit related to ongoing health issue (n=506) | |
| No | 288 (56.9) |
| Yes | 176 (34.8) |
| Unsure | 42 (8.3) |
| **Interventions Received (n=514)** | |
| Labs/bloodwork | 221 (43.0) |
| Oral medications | 220 (42.8) |
| X-ray | 211 (41.1) |
| IV Insertion | 130 (25.3) |
| Consultation with another specialty | 110 (21.4) |
| IV medications | 70 (13.6) |
| ECG^b^ / EEG^c^ (n=1) | 36 (7.0) |
| Medication – other route | 31 (6.0) |
| Inhaled medications | 18 (3.5) |
| In-ED^d^ procedure (laceration repair, lumbar puncture, catheter) | 18 (3.5) |
| Cast application | 10 (1.9) |
| Procedural sedation | 4 (0.8) |
| No interventions received | 60 (11.7) |
| **Final diagnosis (system affected) (n=514)** | |
| Gastrointestinal | 122 (23.7) |
| Musculoskeletal | 115 (22.6) |
| Respiratory | 54 (10.5) |
| Neurologic | 48 (9.3) |
| Genitourinary | 40 (7.8) |
| Head and neck | 37 (7.2) |
| Skin issues | 33 (6.4) |
| Cardiovascular | 26 (5.1) |
| Laceration and trauma | 17 (3.3) |
| Fever and infection | 19 (3.7) |
| Allergy / Immunology / Rheumatology | 9 (1.8) |
| Psychiatric | 7 (1.4) |
| Endocrinology | 3 (0.6) |
| Hematology / Oncology | 3 (0.6) |
| Other | 4 (0.8) |
| **Main spoken language at home (n=504)** | |
| English | 397 (78.8) |
| French | 42 (8.3) |
| Spanish | 10 (2.0) |
| Arabic | 11 (2.2) |
| Hindi / Urdu / Punjabi | 10 (2.0) |
| Portuguese | 4 (0.8) |
| Russian | 4 (0.8) |
| Cantonese | 3 (0.6) |
| Romanian | 2 (0.4) |
| Tagalog | 2 (0.4) |
| Other | 19 (3.7) |
| **Caregiver Highest level of education (n=495) ^e^** | |
| Elementary School | 4 (0.8) |
| Some High School/ High School | 50 (10.1) |
| Diploma / Certificate | 91 (18.4) |
| Some Post-Secondary / University | 105 (21.2) |
| University / Professional Degree | 245 (49.5) |
| **Annual household income (n=422) ^f^** | |
| ≤ $25,000 | 24 (5.7) |
| $25,001 to $50,000 | 65 (15.4) |
| $50,001 to $75,000 | 60 (14.2) |
| $75,000 to $100,000 | 69 (16.4) |
| > $100,000 | 204 (48.3) |

^a^ PRN: pro re nata, as needed

^b^ ECG: electrocardiogram

^c^ EEG: electroencephalogram

^d^ ED: emergency department

^e^ n=6 declined to answer

^f^ n=82 declined to answer
