# Supplementary material for: A national survey of children’s experiences and needs when attending Canadian pediatric emergency departments
Source: PLoS One. 2024 Jun 25;19(6):e0305562. doi: 10.1371/journal.pone.0305562 (PMC11198794; doi:10.1371/journal.pone.0305562)
Supplement: S2 Table — (DOCX) [file pone.0305562.s003.docx]

S2 Table. Thematic coding (n=514)

| **What did we do well today? (n=359)** | **n (%)** |
| --- | --- |
| Good assessment and management of their condition | 129 (35.9) |
| Friendly staff who provided emotional support | 128 (35.7) |
| Staff speaking directly to the child and frequent check ins | 53 (14.8) |
| Timely ED^a^ visit | 36 (10.0) |
| Practical needs such as food, drinks, entertainment and a comfortable environment | 25 (7.0) |
| Everything | 59 (16.4) |
| **What is the ONE most important thing you would tell us to do differently, if you came to the emergency department again? (n=346)** | **n (%)** |
| Shorter wait times | 80 (23.1) |
| Practical needs like food and toys | 51 (14.7) |
| More quiet, private, clean, spacious, comfortable environment | 32 (9.2) |
| More check ins and emotional support | 24 (6.9) |
| Better explanation of ED^a^ processes | 17 (4.9) |
| Better explanation of diagnoses and treatment | 17 (4.9) |
| Better pain and symptom management | 14 (4.0) |
| Nothing else | 90 (26.0) |
| **What would make you happier while waiting? (n=350)** | **n (%)** |
| Electronic entertainment | 175 (50.0) |
| Non-electronic entertainment | 101 (28.9) |
| Food and drinks | 53 (15.1) |
| More comfortable space and comfort items | 51 (14.6) |
| Support persons and animals | 12 (3.4) |
| More check ins with staff | 12 (3.4) |
| Nothing else | 55 (15.7) |
| **What other things did you need, today, to make you more comfortable in the emergency department? (n=321)** | **n (%)** |
| Comfort items like pillows and blankets | 76 (23.7) |
| Entertainment | 64 (19.9) |
| Food and drink | 28 (8.7) |
| Improved physical spaces | 26 (8.1) |
| Support persons and animals | 17 (5.3) |
| More check ins with staff | 13 (4.0) |
| Better pain or medical management | 7 (2.2) |
| Nothing else | 131 (40.8) |

^a^ ED: emergency department
