# Supplementary material for: A national survey of children’s experiences and needs when attending Canadian pediatric emergency departments
Source: PLoS One. 2024 Jun 25;19(6):e0305562. doi: 10.1371/journal.pone.0305562 (PMC11198794; doi:10.1371/journal.pone.0305562)
Supplement: S3 Table — (DOCX) [file pone.0305562.s004.docx]

**S3 Table. Univariable** **model for a child’s understanding of their diagnosis**

| **Independent Variable** | **Odds ratio (95% CI)** | ***p-value*** | ***AUC*** |
| --- | --- | --- | --- |
|  |  |  |  |
| Child age (in years) | 1.09 (1.02, 1.16) | 0.01 ^a^ | 0.57 |
| Previous hospitalizations |  | 0.66 | 0.52 |
| 1-5 vs None | 1.08 (0.71, 1.63) | 0.72 |  |
| 6 or more vs None | 1.53 (0.60, 3.93) | 0.38 |  |
| Main language at home (3 categories) |  | 0.79 | 0.51 |
| French vs English | 0.89 (0.44, 1.78) | 0.73 |  |
| Other vs English | 0.83 (0.48, 1.46) | 0.52 |  |
| Did you feel scared when you first walked into the hospital?  Scared (Likert 3-4-5) vs Not scared (1-2) | 1.02 (0.67, 1.56) | 0.92 | 0.50 |
| Do you feel scared to go home?  Scared (Likert 3-4-5) vs Not scared (1-2) | 0.72 (0.34, 1.52) | 0.39 | 0.51 |
| Did the nurse(s) talk directly to you?  Yes vs No | 2.24 (1.20, 4.19) | 0.01 ^a^ | 0.54 |
| Did the doctor(s) talk directly to you?  Yes vs No | 2.32 (1.24, 4.33) | 0.009 ^a^ | 0.54 |
| Did someone answer your questions and/or worries? |  | 0.01 ^a^ | 0.56 |
| I did not have any questions or worries vs No | 2.26 (1.16, 4.41) |  |  |
| Yes vs No | 2.80 (1.43, 5.49) |  |  |

^a^ Variables with statistical significance < 0.20 were further explored in the multivariable model
