# Supplementary material for: A national survey of children’s experiences and needs when attending Canadian pediatric emergency departments
Source: PLoS One. 2024 Jun 25;19(6):e0305562. doi: 10.1371/journal.pone.0305562 (PMC11198794; doi:10.1371/journal.pone.0305562)
Supplement: S4 Table — (DOCX) [file pone.0305562.s005.docx]

**S4 Table. Univariable model for a child’s understanding of their treatment**

| **Independent Variable** | **Odds ratio (95% CI)** | ***p-value*** | ***AUC*** |
| --- | --- | --- | --- |
|  |  |  |  |
| Child age (in years) | 1.07 (1.01, 1.14) | 0.03 ^a^ | 0.56 |
| Previous hospitalizations |  | 0.56 | 0.51 |
| 1-5 vs None | 0.97 (0.65, 1.45) |  |  |
| 6 or more vs None | 1.65 (0.64, 4.23) |  |  |
| Main language at home (3 categories) |  | 0.83 | 0.51 |
| French vs English | 1.18 (0.58, 2.40) |  |  |
| Other vs English | 1.14 (0.65, 2.01) |  |  |
| Chronic illness |  | 0.48 | 0.52 |
| Unsure vs No | 1.05 (0.50, 2.21) |  |  |
| Yes vs No | 0.75 (0.47, 1.21) |  |  |
| Did you feel scared when you first walked into the hospital?  Scared (3-4-5) vs Not scared (1-2) | 0.56 (0.37, 0.83) | 0.004 ^a^ | 0.56 |
| Do you feel scared to go home?  Scared (3-4-5) vs Not scared (1-2) | 0.38 (0.19, 0.77) | 0.008 ^a^ | 0.53 |
| Did the nurse(s) talk directly to you?  Yes vs No | 1.90 (1.01, 3.58) | 0.046 ^a^ | 0.53 |
| Did the doctor(s) talk directly to you?  Yes vs No | 2.14 (1.14, 4.03) | 0.02 ^a^ | 0.53 |
| Did someone answer your questions and/or worries? |  | 0.007 ^a^ | 0.56 |
| I did not have any questions or worries vs No | 2.55 (1.31, 4.98) |  |  |
| Yes vs No | 2.92 (1.49, 5.70) |  |  |

^a^ Variables with statistical significance < 0.20 were further explored in the multivariable model
