# Supplementary material for: A national survey of children’s experiences and needs when attending Canadian pediatric emergency departments
Source: PLoS One. 2024 Jun 25;19(6):e0305562. doi: 10.1371/journal.pone.0305562 (PMC11198794; doi:10.1371/journal.pone.0305562)
Supplement: S5 Table — (DOCX) [file pone.0305562.s006.docx]

**S5 Table.** **Univariable model for a caregiver’s perception of meeting their child’s needs**

| **Independent Variable** | **Odds ratio (95% CI)** | ***p-value*** | ***AUC*** |
| --- | --- | --- | --- |
|  |  |  |  |
| Previous ED^b^ visits |  | 0.09 ^a^ | 0.54 |
| 1-5 vs None | 0.66 (0.44, 1.001) | 0.051 |  |
| 6 or more vs None | 0.61 (0.39, 0.97) | 0.04 |  |
| Chronic illness |  | 0.55 | 0.52 |
| Unsure vs No | 1.17 (0.67, 2.04) | 0.57 |  |
| Yes vs No | 0.86 (0.61, 1.21) | 0.40 |  |
| Previous hospitalizations |  | 0.90 | 0.51 |
| 1-5 vs None | 0.94 (0.69, 1.28) | 0.68 |  |
| 6 or more vs None | 1.06 (0.53, 2.12) | 0.89 |  |
| Caregiver emotional needs met by ED^b^ staff  Met (4-5) vs Not met (1-2-3) | 8.22 (6.08, 11.11) | <0.0001 ^a^ | 0.74 |
| Child involved in their own care  Met (4-5) vs Not met (1-2-3) | 4.23 (3.18, 5.64) | <0.0001 ^a^ | 0.66 |
| Caregiver involved in child's care  Met (4-5) vs Not met (1-2-3) | 5.90 (4.29, 8.13) | <0.0001 ^a^ | 0.65 |
| Caregiver needs were met  Met (4-5) vs Not met (1-2-3) | 54.27 (36.28, 81.19) | <0.0001 ^a^ | 0.88 |
| Pain management  Met (4-5) vs Not met (1-2-3) | 21.89 (15.64, 30.62) | <0.0001 ^a^ | 0.81 |
| Age of a child (in years) | 0.96 (0.93, 0.98) | 0.002 ^a^ | 0.55 |
| Length of stay (in hours), values over 99h removed | 0.98 (0.97, 0.999) | 0.04 ^a^ | 0.58 |

^a^ Variables with statistical significance < 0.20 were further explored in the multivariable model

^b^ ED: emergency department
